# In Vitro Study of Biological Activity of *Tanacetum vulgare* Extracts

**DOI:** 10.3390/pharmaceutics15020616

**Published:** 2023-02-12

**Authors:** Olga Babich, Viktoria Larina, Olesia Krol, Elena Ulrikh, Stanislav Sukhikh, Maxim A. Gureev, Alexander Prosekov, Svetlana Ivanova

**Affiliations:** 1 Research and Education Center “Industrial Biotechnologies”, Immanuel Kant Baltic Federal University, A. Nevskogo Street 14, Kaliningrad 236016, Russia; 2 Institute of Agroengineering and Food System, Kaliningrad State Technical University, Soviet Avenue 1, Kaliningrad 236022, Russia; 3 Center of Bio- and Chemoinformatics, I.M. Sechenov First Moscow State Medical University, Trubetskaya 8/2, Moscow 119991, Russia; 4 Laboratory of Biocatalysis, Kemerovo State University, Krasnaya Street 6, Kemerovo 650043, Russia; 5 Natural Nutraceutical Biotesting Laboratory, Kemerovo State University, Krasnaya Street 6, Kemerovo 650043, Russia; 6 Department of TNSMD Theory and Methods, Kemerovo State University, Krasnaya Street, 6, Kemerovo 650043, Russia

**Keywords:** *Tanacetum vulgare*, biological activity, extracts, biologically active substances, high-performance liquid chromatography

## Abstract

*Tanacetum vulgare* is an herbaceous plant widely used in folk medicine. It is rich in phenolic acids and flavonoids, which have pharmacological and medicinal properties, such as anthelmintic, antispasmodic, tonic, antidiabetic, diuretic, and antihypertensive. This study aimed to confirm the presence of biologically active substances in *Tanacetum vulgare* and to determine the pharmacological spectrum of biological activity of *Tanacetum vulgare* extract components. When preparing *Tanacetum vulgare* extracts, the highest yield was observed when using the maceration method with a mixture of solvents methanol + trifluoroacetic acid (22.65 ± 0.68%). The biologically active substances in *Tanacetum vulgare* extract samples were determined using high-performance liquid chromatography. Biologically active substances such as luteolin-7-glucoside (550.80 mg/kg), chlorogenic acid (5945.40 mg/kg), and rosmarinic acid (661.31 mg/kg) were identified. Their structures were determined. The experiments have confirmed the antioxidant and antibacterial activities. Secondary metabolites of *Tanacetum vulgare* extracts have been found to have previously unknown biological activity types; experimental confirmation of their existence will advance phytochemical research and lead to the development of new drugs.

## 1. Introduction

The name *Tanacetum vulgare* L., also known as Common Tansy, is derived from the Greek word “athanasia”, which means “immortality”, most likely as a result of the fact that the flowers of this plant do not wilt when dried [1]. This perennial herbaceous plant is widely distributed in North America, Europe, Asia, China, Japan, North Korea, and Russia. *Tanacetum vulgare* L. has been found growing wild in many states of the United States, Europe, and Asia along roadsides, in wastelands, and as a hedge [2,3]. The chemical composition of the plant and its essential oils is affected by its growing environment and climate. It is well described in the literature for many plants [2]. The plants with sectile leaves grow in Corsica (France), Sardinia, and Sicily (Italy), and some consider them to be a separate species (ssp. *siculum*) [4,5].

*T. vulgare* is a 50–100 cm tall perennial herbaceous plant. The stem is straight, branched from the middle, furrowed, glabrous, or slightly pubescent. The leaves are alternate, dark green on top, grayish on the bottom, and pubescent. The lower leaves are short-petiolate, the rest are sessile. All the leaves are pinnately lobed, divided into lanceolate lobes with saw-toothed edges. Anthodia are numerous, 5–8 mm in diameter, and arranged in corymbose inflorescences at the top of the stem. All the flowers are yellow and funnel shaped. The marginal flowers are pistillate, uniseriate; the middle flowers are teleianthous. The fruits are oblong gray achenes 1.5–3 mm long, with five ribs. The plant blooms from mid-June to September and the fruits ripen in August–September [6]. A strong scent comes from the main oil-containing glands in the leaves and flowers [7]. *T. vulgare* is diploid (2n = 18) according to cytogenetic studies [8].

The average amount of essential oil found in aerial parts of *T. vulgare* plants collected at the full flowering stage is from 0.1 to 0.5% [9,10], though up to 1.9% has occasionally been observed [11]. *T. vulgare* essential oil is a warm, slightly spicy, dry, and grassy-smelling liquid with a from yellowish to orange hue. The taste is very pungent and bitter. This plant cannot be used in flavorings or any food products. The essential oil of *T. vulgare* mainly contains high amounts of thujone, a poison that can cause convulsions, vomiting, and uterine bleeding [9]. In addition to the essential oil, *T. vulgare* contains amarines and sesquiterpene lactones [12].

The qualitative and quantitative composition of plant essential oils depends on environmental factors. The content of essential oils is an indirect indicator of plant adaptation [13]. For example, the essential oil of *T. vulgare* growing in Lithuania contains only four main compounds (1,8-cineole, trans-thujone, camphor, and myrtenol) [14], while the essential oil of the *T. vulgare* plant growing on the territory of Scandinavia and the Baltic states [7,15] has a more diverse composition, which includes 15 chemical compounds (α-thujone, β-thujone, camphor, chrysanthenylacetate/chrysanthenol, chrysanthenon, ketone/alcohol, and 1,8-cineole) [11,15]. It was demonstrated that such compounds as β-thujone, camphor, and chrysanthenium acetate are the main components of *T. vulgare* essential oil. These substances were found in the essential oil of *T. vulgare* from 11 different localities in different parts of the world [16,17]. It was noted that the leaves and inflorescences of the *T. vulgare* plant synthesize the same amount of essential oil, but of different composition. The amount of 1,8-cineole in the oil of the whole leaf is higher than in the oils of inflorescences [14].

The phytochemical analysis of the *T. vulgare* plant revealed that it was high in biologically active flavonoids, phenolic compounds, carotenoids, and their derivatives, all of which have been shown to be effective alternative dyes [18]. *T. vulgare* is widely used in traditional medicine. Tea is used as an anthelmintic, carminative, antispasmodic, stimulant of the abdominal organs, tonic, emmenagogue, antidiabetic, diuretic, and antihypertensive agent [19]. In addition, *T. vulgare* is often used in balms, cosmetics, dyes, insecticides, natural preservatives, and medicines [20]. New drugs are discovered by screening a large number of recently synthesized compounds, selecting only those for long-term biomedical analyses that are more effective for their intended purpose and have the fewest negative side effects [21].

This study aimed to confirm the presence of biologically active substances in *T. vulgare* and to determine the pharmacological spectrum of biological activity of *T. vulgare* extract components. It is relevant to have the ability to directly assess the physicochemical characteristics using the biologically active substance (BAS) extracts of *T. vulgare* structural formula.

## 2. Materials and Methods

### 2.1. Reagents

The chemical reagents used in the studies had a purity of at least 95.0% and were of analytical grade or higher (Sigma-Aldrich Rus, Moscow, Russia). The solutions were prepared in deionized water purified with a MilliQ system (MilliporeSigma, Burlington, WY, USA).

### 2.2. T. vulgare Samples for Extraction

The *T. vulgare* plants were collected on the territory of the Kaliningrad region (Russia) in the period June–August 2021. The *T. vulgare* plant samples were collected manually. The plants were dried under natural conditions and collected in paper bags. The plant species were determined under laboratory conditions at the Institute of Living Systems of the I. Kant Baltic Federal University (protocol No. 10-11/2021). The mature *T. vulgare* plants (flowers) were used to study the chemical composition and to produce a plant extract.

### 2.3. Preparation of T. vulgare Extracts

When analyzing the content of biologically active substances (BAS) in *T. vulgare*, the Soxhlet method was used to extract them using methanol, methanol in an acidic medium, and methanol in an alkaline medium over a wide pH range. The extraction module was 1:40 for 15 cycles over a period of up to 8 h. The formic and trifluoroacetic (TFA) acids were used to acidify the medium. The NaOH and NH_4_OH solutions were used for alkalization [16]. According to the literature, the elevated temperature used in the Soxhlet method destroys non-heat-resistant substances [22]. In this regard, maceration was performed at room temperature for 8 h, with stirring and without changing the extraction module (1:40).

After the extraction and maceration steps, 6 kinds of *T. vulgare* extract samples were obtained: (1) methanol Soxhlet extraction; (2) methanolic maceration; (3) methanolic maceration at pH = 12.5 in an alkaline medium with 0.1 N NaOH; (4) methanolic maceration at pH = 10.9 in 0.1 N NH_4_OH solution; (5) methanolic maceration in a solution of 0.1 N trifluoroacetic acid at pH = 1.2; and (6) methanolic maceration in 0.1 N formic acid solution at pH = 3.2. 

The extraction using the Soxhlet method was performed for 8 h (15 cycles). The hydromodulus was 1:40, and the extraction by maceration was performed for 8 h at a temperature of 22 °C with constant stirring.

### 2.4. Determination of BASs in Extracts of T. vulgare

The BASs of *T. vulgare* extracts for bioanalysis were isolated from crude *T. vulgare* flower extracts. The BAS standard samples (luteolin-7-O-glucoside, chlorogenic acid, and rosmarinic acid) for the comparison and identification of these compounds in extracts were purchased from AG Analytekspert (Moscow, Russia). 

The samples of the *T. vulgare* extracts were analyzed using a Shimadzu Prominence LC-20AB chromatograph (Shimadzu, Kyoto, Japan) equipped with a binary pump and an SPD-M20A detector array (Shimadzu, Kyoto, Japan) using high performance liquid chromatography (HPLC). A Zorbax 300SB-C18 4.6 × 250 mm 5 µm column (Agilent, Santa Clara, CA, USA) was also used. The separation temperature in the gradient elution mode was 40 °C. The mobile phase consisted of eluent A (0.1% TFA solution) and eluent B (acetonitrile). A sample (5 μL) was examined at 254, 280, and 325 nm wavelength and a flow rate of 1 mL/minute. 

The components were identified using the spectra of standard samples: chlorogenic acid (chlorogenic acid, CAS 327-97-9, ≥95.0%), rosmarinic acid (rosmarinic acid, CAS 20283-92-5, 96.0%), and luteolin-7-glucoside (cynaroside) (luteolin-7-O-glucoside, CAS 5373-11-5, analytical standard). All the standards and reagents were purchased from AG Analytekspert (Moscow, Russia) with a purity no less than chemical purity. Calibration curves were used to calculate the concentration of biologically active substances in *T. vulgare* extracts (error 3–7%).

### 2.5. Determination of the Antioxidant Activity of BASs from T. vulgare Extracts

The antioxidant activity (AOA) of the BASs from extracts of *T. vulgare* was studied using three methods: a method based on the ability to trap free radicals DPPH (2,2-diphenyl-1-picrylhydrazyl); a method based on the ability to trap free radicals ABTS (2,2/-azino-bis(3-ethylbenzothiazoline-6-sulfonic acid); and a method based on the restoring power upon interaction with the Fe(III)-2.4.6-tripyridyl-s-triazine (FRAP) complex. 

DPPH is a stable free radical that reacts with the hydrogen atom released by the substrate. The antioxidant activity was assessed by the presence or absence of a dark purple color of the DPPH solution and absorption bands in the ethanol solution at a wavelength of 517 nm using a CLARIOstar microplate reader (BMG Labtech, Ortenberg, Germany).

To prepare a solution of ABTS radicals, an aliquot of a solution of ABTS with a concentration of 7.0 mmol/L and a solution of potassium persulfate with a concentration of 2.45 mmol/L were mixed and incubated in a dark place at 25 °C for 16 h. Then, 20 μL of BASs or a standard sample was added to 300 μL of the prepared solution of the ABTS radical cation. The mixture was incubated for 15 min at 37 °C in the dark and the optical density was determined at 734 nm on a UV-1280 spectrophotometer (Shimadzu, Kyoto, Japan). 

The antioxidant activity of the biologically active substances was studied using a FRAP solution. For this, one part of a 10 mmol/L solution of 2,4,6-tripyridyl-s-triazine in an HCl solution with a concentration of 40 mmol/L, one part of a FeCl_3_ × 6H_2_O solution with a solution concentration of 20 mmol/L, and 10 parts of an acetate buffer with concentration of 0.3 mol/L (pH = 3.6) were mixed [23]. The reaction was performed at 37 °C in the dark for 10 min. The light absorption was measured at 593 nm on a UV-1280 spectrophotometer (Shimadzu, Kyoto, Japan). 

### 2.6. Determination of the Antimicrobial Activity of BASs from T. vulgare Extracts

The antimicrobial properties of biologically active substances were studied using the disk-diffusion method against test strains of *Bacillus subtilis* (Gram-positive bacterium), *Escherichia coli*, *Pseudomonas aeruginosa* (Gram-negative bacteria), *Candida albicans* (yeast). *E. coli*, *P. aeruginosa*, and *B. subtilis* strains were cultivated on solid and liquid LB nutrient mediums (Dia-M, Moscow, Russia) at 37 °C. The strains of microscopic fungi *C. albicans* were cultivated on a Ringer’s nutrient medium (Dia-M, Moscow, Russia) at a temperature of 25 °C. The following concentrations were used to determine antimicrobial activity: 0.625, 1.25, 2.5, 5, 10, 25, 50, 100, 150, and 200 µg/disk. An antibiotic (kanamycin) at a concentration of 50 µg/disk (for bacteria) and fluconazole at a concentration of 500 µg/disk (for yeast-like fungi—*C. albicans*) were used as a positive control. A solution of TFA (31%) and acetonitrile (69%) was used as a negative control.

Three parallel measurements were recorded. The measurement result was selected as the mean value.

### 2.7. Molecular Docking

In the Schrödinger software package for selected antibiotic activity target proteins, the molecular modeling of binding to isolated natural compounds was performed. The molecular modeling algorithm was standard and consisted in the preparation of selected target proteins, preparation of ligands, subsequent docking, and evaluation of target binding. The compounds are ranked in scoring function values as Gibbs binding energies. The crystal structures of all the targets were downloaded from RCSB PDB database (https://www.rcsb.org/ accessed on 9 February 2023) with corresponding PDBID—transcription initiation complex (6VVT) [24], dihydrofolate reductase (2WV3) [25], elongation factor G (2BV3) [26], enoyl-acyl carrier protein reductase (2PD4) [27], and deacetylase LpxC (2GO4) [28]. All the ligands, ions, and water molecules were removed from the structures. Hydrogen was first added to each structure, and then the polar hydrogen atoms were removed. The compound library was prepared in the LigPrep module. Molecular docking was performed using the Schrödinger software package (Schrödinger, LLC: New York, NY, USA, 2017) using the gelid algorithm with the corresponding scoring function calculated [29,30].

### 2.8. Statistical Data Processing

The obtained data were statistically processed using the SigmaPlot 12.3 program (Systat Software GmbH, Erkrath, Germany). The obtained indicators were expressed as the mean value ± standard deviation. All the experiments were repeated three times. A one-way ANOVA was used to determine statistically significant differences between the mean values. Using the Shapiro–Wilks test and uniformity of variance, the data were tested for normal distribution before ANOVA analysis. The Tukey test was used to find the differences between the data’s mean values at a significance level of *p* < 0.05.

## 3. Results

The maximum yield of dry extracts was determined in the experiment. Table 1 shows the total yield of *T. vulgare* plant extracts.

Since the highest extract yield (22.65 ± 0.68%) was observed when using the method of maceration of *T. vulgare* with a mixture of methanol and trifluoroacetic acid (TFA). Then, HPLC was used to study the composition of all the obtained extracts (Table 2). Among the methanolic extracts, the highest yield was observed during extraction using the Soxhlet method; therefore, this extract was subjected to further study.

Table 2 lists the BASs identified in the *T. vulgare* extract samples.

**Figure 1:**
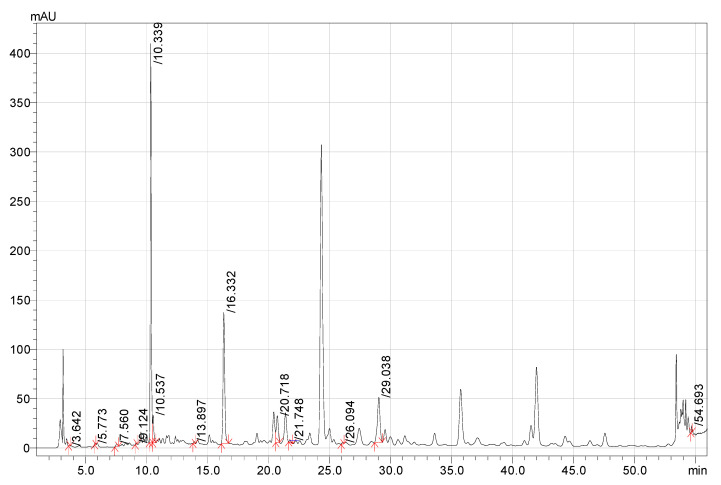
HPLC chromatogram of *T. vulgare* extract samples (Methanol–TFA).

The extract producing methanol with trifluoroacetic acid had the highest concentration of the phenolic components. This extract was selected for research on biological activity. All the other identified BASs that are demonstrated in the chromatogram (Figure 1) were found in trace amounts. In addition to the components listed in Table 2, the chromatogram of this extract showed unidentified peaks of high intensity (24.3 min, 35.7 min, and 41.5 min); the absorption spectra of which corresponded to phenolic acids. Further research is needed to identify these components.

The HPLC chromatogram of the *T. vulgare* methanol extract with 0.1 N TFA is presented in Figure 1.

**Figure 2:**
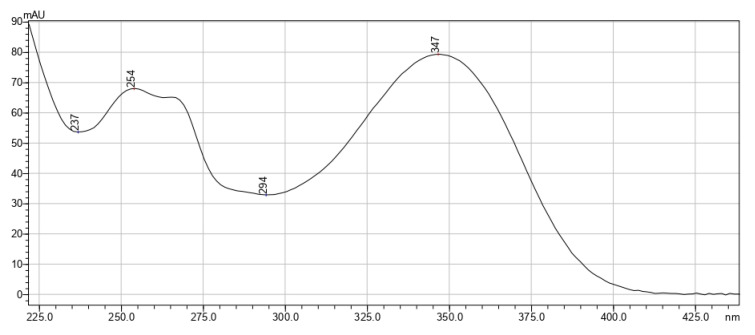
Absorption spectrum of luteolin-7-glucoside isolated from *T. vulgare* extracts.

**Figure 3:**
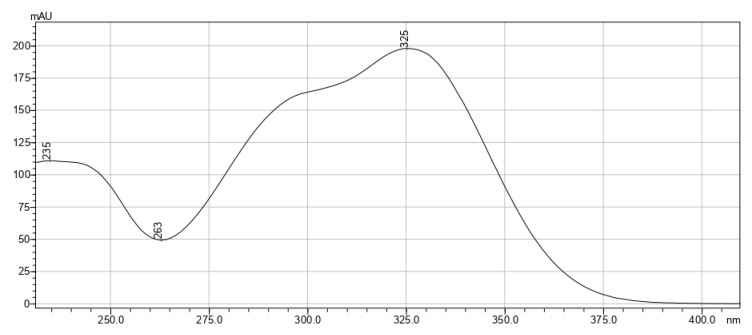
Absorption spectrum of chlorogenic acid isolated from *T. vulgare* extracts.

**Figure 4:**
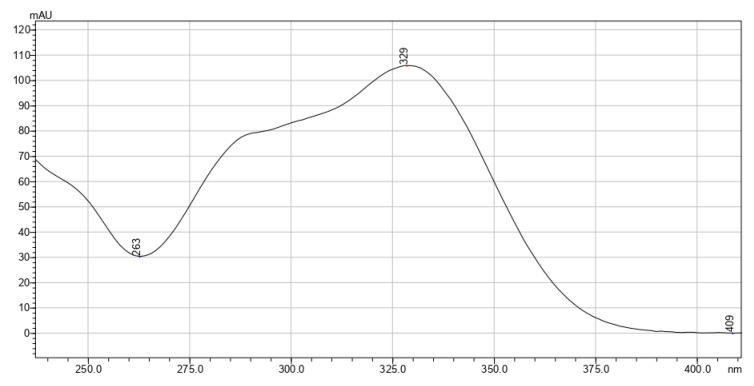
Absorption spectrum of rosmarinic acid isolated from *T. vulgare* extracts.

Figure 2, Figure 3 and Figure 4 exhibit the absorption spectra of the main isolated biologically active substances from the extracts.

**Figure 5:**
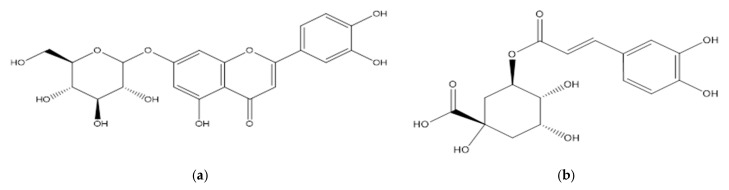

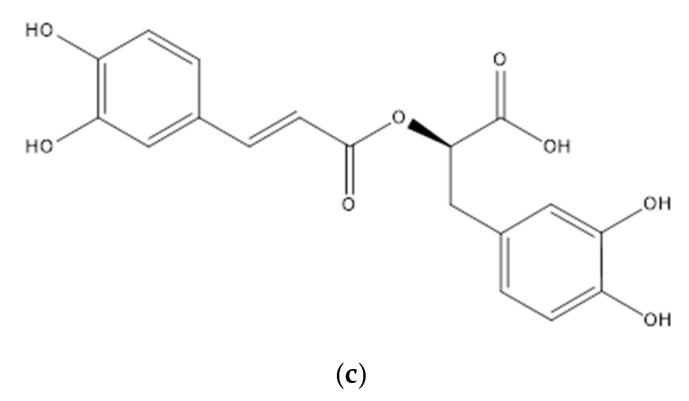
Structure of BASs from *T. vulgare* extracts: (**a**) luteolin-7-glucoside; (**b**) chlorogenic acid; (**c**) rosmarinic acid.

Figure 5 shows the structural formulas of the main HPLC-identified (by retention time and absorption spectra) BASs from *T. vulgare* extracts.

The predetermined BAS detection threshold is small (about 20%), which indicates a rare structure of the compound for the predicted activities. We focused on the three main components of *T. vulgare* extracts in this study: lutelin-7-glucoside, chlorogenic, and rosmarinic acids, because they are found in the greatest quantities in the *T. vulgare* extracts and have the highest antioxidant and antimicrobial activity when compared to other identified biologically active substances (3,4-dihydroxybenzoic acid, neochlorogenic acid, caftaric acid, caffeic acid, coumaric acid, ferulic acid, and apigenin-7-O-glucoside). Lutelin-7-glucoside, chlorogenic, and rosmarinic acids are well-known compounds with numerous research papers describing their properties [31,32,33].

The simulation results show a high binding capacity of the natural compounds found in the *T. vulgare* extracts to the selected targets. For each target, natural compounds capable of binding were found. The highest binding properties are: Neochlorogenic acid, Luteolin-7-glucoside, Apigenin-7-O-glucoside, and Chlorogenic acid. The following enzymes have the most sensitive binding: enoyl-acyl carrier protein reductase, which is prone to the selective action of Luteolin-7-glycoside and Apigenin-7-O-glucoside, and deacetylase LpxC, which selectively binds neochlorogenic, chlorogenic, and cafftaric acids. The high selectivity of binding of caftaric acid to elongation factor G (2BV3) is noticeable. From the modeling results, it can be concluded that the potentiation of the 2PD4 and 2GO4 enzymes can lead to an antibacterial effect against gamma-positive and Gram-negative bacteria. The results of molecular docking are in good agreement with the literature data on the study of the potentiation of the enzymatic targets of our choice using antibacterial drugs with new mechanisms of action [34,35,36]. Table 3 presents the scoring function (Gibbs energy in kcal/mol) to the corresponding compound end target.

**Figure 6:**
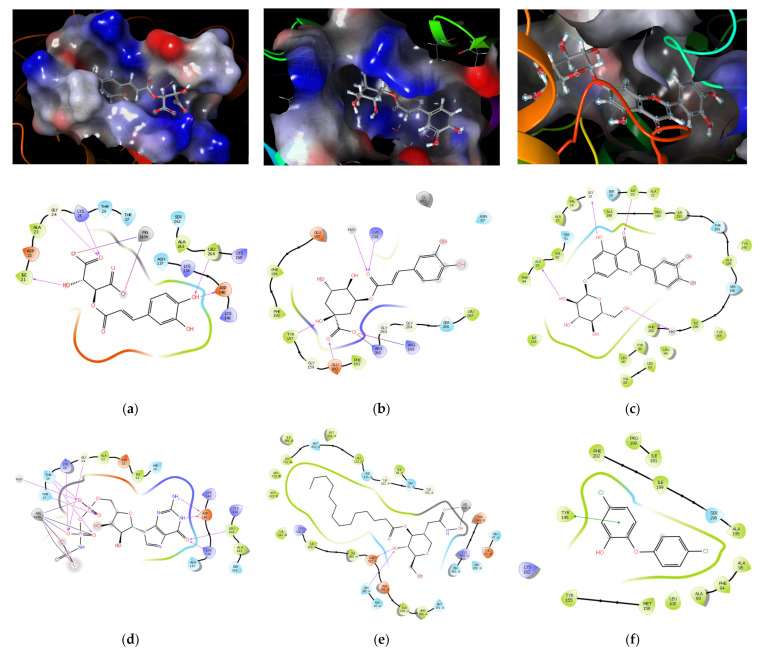
Binding conformations after molecular docking for the most potent targets and ligands: (**a**) Rosmarinic acid, the diagram shows the formation of a salt bridge with a magnesium ion, as well as the interaction with the electrostatic surface of the binding site with 2BV3; (**b**) chlorogenic acid, hydrogen bond diagram and electrostatic binding site surface with 2GO4; (**c**) luteolin-7-O-glycoside, hydrogen bond diagram and electrostatic surface of the binding site with 2PD4; (**d**) ligand interaction diagram for 2BV3 with natural ligand; (**e**) ligand interaction diagram for 2GO4 with natural ligand; and (**f**) ligand interaction diagram for 2PD4 with natural ligand.

Figure 6 depicts the interaction of amino acid side chains of binding sites with most potent ligands and their targets.

The antioxidant and antimicrobial properties of biologically active substances in *T. vulgare* extracts were studied to confirm the biological activity. The antioxidant activity (AOA) of the BASs from the *T. vulgare* extracts was assessed using three different methods to produce more convincing results: the capacity to bind free radicals DPPH and ABTS, as well as the reducing power when interacting with the Fe (III) complex (FRAP). Six crude extracts of *T. vulgare* were obtained for analysis in the first stage, and individual biologically active substances of these extracts were isolated: lutelin-7-glucoside, chlorogenic, and rosmarinic acids, and compared with pure, standard samples of lutelin-7-glucoside, chlorogenic, and rosmarinic acids purchased for analysis. Table 4 presents the results of studying the antioxidant activity of individual biologically active substances isolated from *T. vulgare* extracts (lutelin-7-glucoside, chlorogenic, and rosmarinic acids).

The results of determining the antimicrobial activity of BASs from *T. vulgare* extracts are presented in Table 5.

## 4. Discussion

The following BASs were mainly identified using HPLC in samples of *T. vulgare* extract, produced using the maceration method with a mixture of methanol–TFA solvents: luteolin-7-glucoside (C_21_H_20_O_11_)—550.80 mg/kg, chlorogenic acid (C_16_H_18_O_9_)—5945.40 mg/kg, and rosmarinic acid (C_18_H_16_O_8_)—661.31 mg/kg. 3,4-dihydroxybenzoic, neochlorogenic acid, caffeic, coumaric, ferulic, and cafftaric acids, acacetin, and apigenin-7-O-glucoside were detected either in minimal or trace amounts. 

It is known [37] that different classes of phenolic compounds exhibit different antioxidant activity measured in vitro with respect to 2,2-diphenyl-1-picrylhydrazyl and 2,2/-azino-bis(3-ethylbenzothiazoline-6-sulfonic acid) radicals, and also differ in the ability to reduce Fe (III) in a complex with 2.4.6-tripyridyl-s-triazine. Chlorogenic and rosmarinic acids, a subclass of hydroxycinnamic acids, were isolated from *T. vulgare* extracts. Chlorogenic acid had the maximum antioxidant activity measured by all three methods (12.31 ± 0.98, 7.52 ± 0.32, and 6.91 ± 0.22 mmol Trolox equivalent/g measured using ABTS, DPPH, and FRAP methods, respectively). Rosmarinic acid, also belonging to the class of hydroxycinnamic acids and Isolated from *T. vulgare* extracts, showed approximately 2 times lower antioxidant activity compared to chlorogenic acid (5.69 ± 0.29, 3.44 ± 0.19, and 3.74 ± 0.12 mmol Trolox equivalent/g according to the ABTS, DPPH, and FRAP methods, respectively) [38]. 

The results show that in vitro antimicrobial activity of 5 µg/disk of luteolin-7-glucoside against *B. subtilis, P. aeruginosa,* and *E. coli* and 10 µg/disk of luteolin-7-glucoside against *C. albicans*; 5 µg/disc of chlorogenic acid exhibited antimicrobial activity against *B. subtilis, P. aeruginosa,* and *E. coli* but no antimicrobial activity against *C. albicans*; 5 µg/disk of rosmarinic acid exhibits antimicrobial activity against *B. subtilis, P. aeruginosa,* and *E. coli*, but not against *C. albicans*.

The data above demonstrated that the wide range of pharmacological effects and mechanisms of action that the BASs from *T. vulgare* extracts can have was confirmed by the diversity of the biological activities of these BASs. Particularly, several activities indicated that investigations into the potential use of these compounds as pharmaceutical ingredients with previously unknown antimutagenic, antihypoxic, antioxidant, antibacterial, and cardioprotective actions are promising. In the case of synergism or antagonism of the combined action of biologically active substances in real extracts, in vitro and in vivo experiments are required to confirm the presence of the activities [39,40,41].

To study the mechanism of antioxidant and antibacterial properties, Filimonov et al. [39] studied the processes of molecular docking of carvacrol with xanthine oxidase enzyme and antibacterial protein. In the course of the study, the author proved that the xanthine oxidase enzyme is actively involved in the formation of reactive oxygen species, and the carrier protein reductase is a target for inhibiting bacterial growth [39].

A group of scientists led by Kurashov [42] proved that certain compounds, such as androstanol; biform; geranilacetone; kauren; manul; muketon; rimouin; sandaracopymaradiene; α-eudesmol; α-muurolene; β-eudesmol; β-ionone; (22E)-3α-ergosta-14.22-dien-5β-ol acetate; (2Z,4Z)-hepta-2,4-dienal; (3E,5E)-octa-3,5-diene-2-one; (9Z,12Z)-octadeca-9.12-dienoic acid; 2,6-dimethylcyclohexan-1-ol; 2-phenylacetaldehyde; 5α—androstan-16-one; and 8-(2.5.5.8A-tetramethyl-1.4.4A.5.6.7.8.8A-octahydro-1-naphthalenyl)-6-methyl-5-octen-2-ol, have pronounced antitumor, antimicrobial, and anti-inflammatory properties, which indicates the high potential for using plant extracts containing these substances in the pharmacological industry.

In a study [15], extracts of *T. vulgare* inflorescences were obtained, which were distinguished by their high (8.8 mmol Trolox equivalent/g) antioxidant activity and determined by the value of their DPPH inhibition. The obtained values of the antioxidant activity of the *T. vulgare* extracts correlate well with the total content of the phenolic acids [11,21]. These results confirm that the presence of chlorogenic and rosmarinic acids affect the antioxidant activity of extracts of *T. vulgare* inflorescences. The presence of antioxidant activity of *T. vulgare* plant extracts confirm the high probability of antioxidant activity of individual BASs isolated in our studies. The difference in the values of antioxidant activity obtained in our study and the values of antioxidant activity obtained in the study [15] is explained by different habitats of *T. vulgare*. 

The research by Devrnja et al. [1] enabled it to be possible to establish that the extract of *T. vulgare* has the ability to inhibit biofilm synthesis and also exhibits antioxidant and antimicrobial properties. The same research group showed the possibility of using *T. vulgare* extract in the pharmaceutical and cosmetic industries [1]. 

A study [43] aimed to determine the antibacterial and antioxidant activities of *T. vulgare* hydroethanol extracts based on their chemical profiles. The dominant compound of *T. vulgare* extracts, determined using gas chromatography with mass spectrometry, was trans-chrysanthenyl acetate (18.39%). HPLC-DAD was used to determine the chemical composition of phenolic acids and flavonoids. Chicoric acid was the dominant phenolic compound (4311.3 mg × 100 g^−1^). The of *T. vulgare* extract had a high antioxidant potential (determined using FRAP and DPPH). 

The plant extracts have been found to have antioxidant and antimicrobial properties, causing them to be promising raw materials for the pharmaceutical and food industries. These properties were also confirmed in studies by Bączek et al. [43]. They found that extracts of *T. vulgare* are characterized by antibacterial, fungicidal, and antioxidant properties. The phytochemical analysis of plant extracts revealed the presence of flavonoids (luteolin, quercetin, and apigenin) and phenolic acids (dicaffeoylquinic acid, caffeic acid, and chlorogenic acid) [43].

In a study [44], *T. vulgare* essential oil extracts demonstrated antioxidant activity, significantly inhibiting tert-butyl hydroperoxide-induced DCFH oxidation. Sharopov et al. [44] proved that thymol, carvacrol, and eugenol, which belong to the terpenoid series, have antioxidant, antimicrobial, and antifungal activity [45]. It was established that the α-pinene and caryophyllene oxide compounds contained in extracts of *T. vulgare* have the highest antioxidant activity [46]. The paper [47] described moderate levels of α-pinene antioxidant activity based on DPPH analysis. However, in [47], the antioxidant activity of caryophyllene oxide was reported for the first time.

As reported in [48], *T. vulgare* extracts have antibacterial activity [48,49,50]. The antibacterial activity of *T. vulgare* from Romania [51] and Slovakia [52] against strains of Gram-positive bacteria using the disk diffusion method has also been reported. The extracts of the essential oil of *T. vulgare* from Tajikistan were found to be weakly effective against *E. coli* and MRSA [44]. The studies show that *T. vulgare* essential oil extracts are effective against *S. aureus* and *E. coli*. In addition, the caryophyllene oxide and γ-terpinene extracts were also active against *S. aureus* [53]. The antibacterial activity of caryophyllene oxide and γ-terpinene from *T. vulgare* extracts has been previously reported in the literature [54,55].

## 5. Conclusions

The following BASs were mainly identified using HPLC in samples of *T. vulgare* extract produced using the maceration method with a mixture of methanol–TFA solvents: luteolin-7-glucoside (C_21_H_20_O_11_)—550.80 mg/kg, chlorogenic acid (C_16_H_18_O_9_)—5945.40 mg/kg, and rosmarinic acid (C_18_H_16_O_8_)—661.31 mg/kg. Additionally, 3,4-dihydroxybenzoic, caffeic, p-coumaric, chicoric, and cafftaric acids, and quercetin-3D-glucoside were found in minimal and trace amounts. 

It has been proven through in vitro experiments that *T. vulgare* extracts can exhibit a wide range of activities because of the presence of luteolin-7-glucoside, chlorogenic, and rosmarinic acids, but the most likely ones are antimutagenic, antihypoxic, antioxidant, antibacterial, and cardioprotective activities. This study also demonstrated that the dominant components (luteolin-7-glucoside, chlorogenic, and rosmarinic acids) have antioxidant and antimicrobial activity. Previously unknown types of biological activity of *T. vulgare* extract secondary metabolites have been identified, and experimental confirmation of their presence will contribute to the advancement of phytochemical research and the development of new drugs.

## Figures and Tables

**Table 1:** Total yield of *T. vulgare* extracts.

Name	Extract Yield, wt. %					
	Soxhlet Method	Maceration Method				
	Methanol	Methanol	Methanol–NaOH	Methanol–NH_4_OH	Methanol–TFA	Methanol–HCOOH
*T. vulgare*	17.10 ± 0.51 ^a^	8.98 ± 0.27 ^b^	4.56 ± 0.13 ^c^	18.74 ± 0.56 ^a^	22.65 ± 0.68 ^d^	17.25 ± 0.51 ^a^

Values in row followed by the same letter do not significantly differ (*p* < 0.05) as assessed using the post hoc test (Duncan’s test). Data presented as a mean ± SD (*n* = 3).

**Table 2:** Amount of biologically active substances identified in *T. vulgare* extracts.

BAS	Retention Time, min	Content, mg/kg		
		Methanol–TFA	Methanol	Methanol–NaOH
3,4-dihydroxybenzoic acid	5.8 ± 0.5	14.40 ± 0.43	15.36 ± 0.46	-
Neochlorogenic acid	7.6 ± 0.5	112.14 ± 3.36	-	-
Caftaric acid	9.1 ± 0.5	63.00 ± 1.89	- *	-
Chlorogenic acid	10.3 ± 0.5	5945.40 ± 178.36	2265.24 ± 67.96	-
Caffeic acid	10.5 ± 0.5	280.80 ± 8.42	93.45 ± 2.80	- *
Coumaric acid	13.9 ± 0.5	- *	- *	- *
Ferulic acid	16.3 ± 0.5	1818.00 ± 54.54	-	- *
Luteolin-7-glucoside	20.7 ± 0.5	550.80 ± 16.52	104.88 ± 3.15	-
Chicoric acid	21.7 ± 0.5	- *	80.05 ± 2.40	-
Apigenin-7-O-glucoside	26.1 ± 0.5	30.60 ± 0.91	-	-
Rosmarinic acid	29.3 ± 0.5	1764.00 ± 52.92	661.31 ± 19.84	3.72 ± 0.11
Acacetin	54.7 ± 0.5	23.40 ± 0.70	-	-

* Below detection limit. Data presented as a mean ± SD (*n* = 3).

**Table 3:** Scoring function for corresponding enzyme and natural product.

Compounds	6VVT	2WV3	2BV3	2PD4	2GO4
3,4-dihydroxybenzoic acid	2.90	5.29	6.75	5.42	3.07
Neochlorogenic acid	6.54	9.02	7.71	10.22	9.14
Cafftaric acid	5.88	7.65	9.65	8.66	8.37
Chlorogenic acid	5.77	9.49	6.15	9.77	9.93
Caffeic acid	3.68	5.67	5.38	4.66	1.35
Coumaric acid	-	-	-	-	8.22
Ferulic acid	2.96	5.58	-	5.02	4.47
Luteolin-7-glucoside	8.20	11.46	7.12	12.55	7.57
Apigenin-7-O-glucoside	6.27	10.34	7.32	12.12	8.00
Rosmarinic acid	5.49	8.92	9.29	8.94	5.59
Acacetin	2.23	5.68	3.64	4.80	3.97

**Table 4:** Antioxidant properties of BASs from *T. vulgare* extracts.

Active Ingredients	Antioxidant Activity, mmol Trolox Equivalent/g		
	ABTS	DPPH	FRAP
Luteolin-7-glucoside	6.5 ± 0.34	2.99 ± 0.16	1.79 ± 0.06
Chlorogenic acid	12.31 ± 0.98	7.52 ± 0.32	6.91 ± 0.22
Rosmarinic acid	5.69 ± 0.29	3.44 ± 0.19	3.74 ± 0.12

Data presented as a mean ± SD (*n* = 3).

**Table 5:** Antimicrobial activity (diameter of the growth inhibition zone of test microorganisms, mm) of BASs from *T. vulgare* extracts.

Active Components	BAS Weight, µg/disk									
	200	150	100	50	25	10	5	2.5	1.25	0.625
	*B. subtilis*									
Luteolin-7-glucoside	–	–	–	6.0 ± 0.2 ^a^	8.0 ± 0.2 ^b^	8.0 ± 0.2 ^b^	10.0 ± 0.3 ^b^	7.0 ± 0.2 ^ab^	3.0 ± 0.1 ^c^	–
Chlorogenic acid	–	–	–	4.0 ± 0.1 ^a^	5.0 ± 0.2 ^a^	6.0 ± 0.2 ^ab^	8.0 ± 0.2 ^b^	6.0 ± 0.2 ^ab^	2.0 ± 0.1 ^a^	–
Rosmarinic acid	–	–	–	3.0 ± 0.1 ^a^	4.0 ± 0.1 ^a^	6.0 ± 0.2 ^ab^	7.0 ± 0.2 ^b^	5.0 ± 0.2 ^a^	2.0 ± 0.1 ^a^	–
	*P. aeruginosa*									
Luteolin-7-glucoside	–	–	–	5.0 ± 0.2 ^a^	6.0 ± 0.2 ^a^	7.0 ± 0.2 ^ab^	8.0 ± 0.2 ^b^	5.0 ± 0.2 ^a^	2.0 ± 0.1 ^c^	–
Chlorogenic acid	–	–	–	6.0 ± 0.2 ^a^	6.0 ± 0.2 ^a^	7.0 ± 0.2 ^a^	7.0 ± 0.2 ^a^	3.0 ± 0.1 ^b^	–	–
Rosmarinic acid	–	–	–	4.0 ± 0.1 ^a^	6.0 ± 0.2 ^ab^	7.0 ± 0.2 ^b^	8.0 ± 0.2 ^b^	6.0 ± 0.2 ^ab^	3.0 ± 0.1 ^a^	–
	*E. coli*									
Luteolin-7-glucoside	–	–	–	1.0 ± 0.1 ^a^	2.0 ± 0.1 ^a^	2.0 ± 0.1 ^a^	5.0 ± 0.2 ^b^	1.0 ± 0.1 ^a^	–	–
Chlorogenic acid	–	–	–	1.0 ± 0.1 ^a^	2.0 ± 0.1 ^a^	3.0 ± 0.1 ^ab^	4.0 ± 0.1 ^b^	2.0 ± 0.1 ^a^	–	–
Rosmarinic acid	–	–	–	1.0 ± 0.1 ^a^	3.0 ± 0.1 ^a^	4.0 ± 0.1 ^ab^	5.0 ± 0.2 ^b^	2.0 ± 0.1 ^a^	–	–
	*C. albicans*									
Luteolin-7-glucoside	–	–	–	–	1.0 ± 0.1 ^a^	6.0 ± 0.2 ^b^	2.0 ± 0.1 ^a^	–	–	–
Chlorogenic acid	–	–	–	–	–	–	–	–	–	–
Rosmarinic acid	–	–	–	–	–	–	–	–	–	–

Values in row followed by the same letter do not differ significantly (*p* < 0.05) as assessed using the post hoc test (Duncan’s test).

## Data Availability

The data are included in the manuscript.
